# Supplementary material for: Presence of N, N′-Substituted p-Phenylenediamine-Derived Quinones in Human Urine
Source: Toxics. 2024 Oct 11;12(10):733. doi: 10.3390/toxics12100733 (PMC11511057; doi:10.3390/toxics12100733)
Supplement: Supplementary file 1 [file toxics-12-00733-s001.zip › toxics-3204458-supplementary.pdf]

SUPPORTING INFORMATION

Presence of *N*, *N*'-Substituted *p*-Phenylenediamine-Derived Quinones in Human Urine

Juxiu Huang[1,#], Hangbiao Jin[2,#], Yingying Zhu[3], Ruyue Guo[2], Lisha Zhou[1], Xiaoyu Wu[1,*]

[1] Taizhou Central Hospital (Taizhou University Hospital), School of Medicine, Taizhou University, Taizhou, Zhejiang 318000, P. R. China.

[2] Key Laboratory of Microbial Technology for Industrial Pollution Control of Zhejiang Province, College of Environment, Zhejiang University of Technology, Hangzhou, Zhejiang 310032, P. R. China.

[3] School of Life Sciences, Taizhou University, Taizhou, Zhejiang 318000, P. R. China.

[#] These two authors contributed equally to this study.

Table S1. List of Target PPDQs in the Present Study and Their Abbreviations and Full Names.

Abbreviation	Full name
**IPPDQ**	2-(isopropylamino)-5-(phenylamino)cyclohexa-2,5-diene-1,4-dione
**DPPDQ**	2,5-bis(phenylamino)cyclohexa-2,5-diene-1,4-dione
**CPPDQ**	2-(cyclohexylamino)-5-(phenylamino)cyclohexa-2,5-diene-1,4-dione
**6PPDQ**	2-((4-methylpentan-2-yl)amino)-5-(phenylamino)cyclohexa-2,5-diene-1,4-dione
**77PDQ**	2,5-bis((5-methylhexan-2-yl)amino)cyclohexa-2,5-diene-1,4-dione
**DTPDQ**	2,5-bis(o-tolylamino)cyclohexa-2,5-diene-1,4-dione

Table S2. Demographic Characteristics of Participants (*n* = 149) Recruited in This Study.

	n (%) or mean ± SD [a]
**Gender**	
Males	67 (45%)
Females	82 (55%)
**Age**	
Males	46 ± 11
Females	47 ± 14
**Age group**	
24−30 years	15 (10%)
31−40 years	42 (28%)
41−50 years	45 (30%)
51−62 years	47 (32%)
**BMI (kg/m^2)**	
Males	28 ± 6.1
Females	24 ± 5.5
**Education level**	
College	20 (14%)
High school	69 (46%)
Below high school	60 (40%)
**Annual household income (CNY)**	
< 80, 000	51 (34%)
80, 000−150, 000	82 (55%)
> 150, 000	16 (11%)

[a] % means the percentage to the total participants, and ± means the +/−.

Table S3. MRM Transition Parameters for PPDQs and Internal Standard.

	MRM transition		Cone voltage (eV)	Collision energy (eV)	SRM ratio (average)
	Parent ion	Daughter ion			
**6PPDQ**	299	215	38	15	4.5
		187	38	27	
**IPPDQ**	257	187	33	28	9.8
		215	33	22	
**DPPDQ**	291	263	38	12	2.2
		170	38	30	
**DTPDQ**	319	301	40	14	2.8
		212	40	22	
**CPPDQ**	297	187	35	28	2.5
		98	35	35	
**77PDQ**	335	237	40	15	4.1
		139	40	28	
**6PPDQ-d_5**	304	192	38	27	

Table S4. LODs and Extraction Recoveries of PPDQs in Human Urine.

	LOD	Extraction Recovery (%)										
		Spiked at 0.5 ng/mL			Spiked at 5.0 ng/mL			Spiked at 50 ng/mL				
	(ng/mL)	mean	SD	Range	mean	SD	Range	mean	SD	Range	Equation	R^2
**IPPDQ**	0.095	82	9.0	71-92	87	10	75-98	87	8.0	80-96	y = 0.36 x -0.022	0.9956
**DPPDQ**	0.017	107	5.2	102-114	105	5.4	98-113	102	7.4	95-110	y = 2.1 x + 0.81	0.9991
**CPPDQ**	0.052	105	10	92-116	92	6.6	86-99	98	10	85-110	y = 1.4 x + 0.66	0.9989
**6PPDQ**	0.033	92	8.6	98-101	86	5.9	83-96	96	5.8	90-105	y = 2.9 x -0.53	0.9978
**77PDQ**	0.081	92	6.3	86-100	87	5.1	81-95	88	5.9	82-94	y = 0.44 x + 0.25	0.9990
**DTPDQ**	0.097	99	8.1	90-107	89	7.3	81-97	99	6.2	92-106	y = 1.9 x + 0.69	0.9979

Table S5. Correlations among Concentrations of Various PPDQs in Human Urine.

		DPPDQ	CPPDQ	77PDQ	6PPDQ	DTPDQ
**IPPDQ**	r_s	.134	.139	.003	.208	.296
	p	.265	.239	.982	.066	.130
**DPPDQ**	r_s	1	.278	.123	.288	.592**
	p		.164	.221	.202	.000
**CPPDQ**	r_s		1	.232	.733**	.267
	p			.115	.000	.117
**77PDQ**	r_s			1	.129	.048
	p				.244	.676
**6PPDQ**	r_s				1	.290
	p					.206

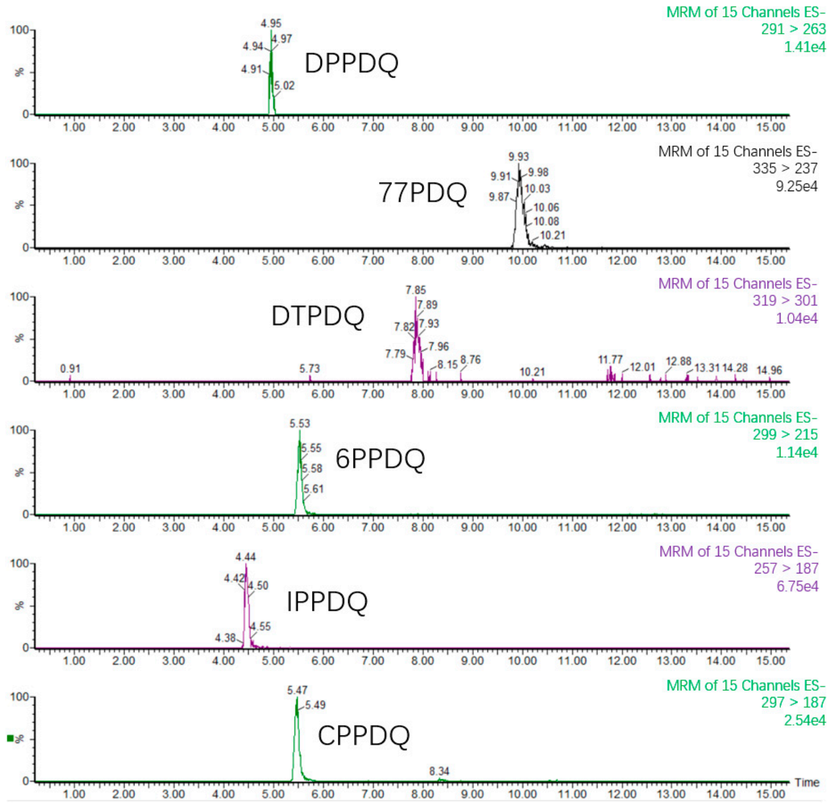

Figure S1. Representative chromatograms of target analytes in the human urine sample.
